# PD-1 Blockade During Post-partum Involution Reactivates the Anti-tumor Response and Reduces Lymphatic Vessel Density

**DOI:** 10.3389/fimmu.2019.01313

**Published:** 2019-06-11

**Authors:** Beth A. Jirón Tamburini, Alan M. Elder, Jeffrey M. Finlon, Andrew B. Winter, Veronica M. Wessells, Virginia F. Borges, Traci R. Lyons

**Affiliations:** ^1^Division of Gastroenterology and Hepatology, Department of Medicine, School of Medicine, University of Colorado Anschutz Medical Campus, Denver, CO, United States; ^2^Department of Immunology and Microbiology, Department of Medicine, School of Medicine, University of Colorado Anschutz Medical Campus, Denver, CO, United States; ^3^Division of Medical Oncology, Department of Medicine, School of Medicine, University of Colorado Anschutz Medical Campus, Denver, CO, United States; ^4^Young Women's' Breast Cancer Translational Program and University of Colorado Cancer Center, Aurora, CO, United States

**Keywords:** lymphatic endothelial cells, post-partum breast cancer, metastasis, immunotherapy, PD-L1, PD-1, T cells

## Abstract

Post-partum breast cancer patients, or breast cancer patients diagnosed within 10 years of last childbirth, are ~3–5 times more likely to develop metastasis in comparison to non-post-partum, or nulliparous, patients. Additionally, post-partum patients have increased tumor-associated lymphatic vessels and LN involvement, including when controlled for size of the primary tumor. In pre-clinical, *immune-competent*, mouse mammary tumor models of post-partum breast cancer (PPBC), tumor growth and lymphogenous tumor cell spread occur more rapidly in post-partum hosts. Here we report on PD-L1 expression by lymphatic endothelial cells and CD11b+ cells in the microenvironment of post-partum tumors, which is accompanied by an increase in PD-1 expression by T cells. Additionally, we observed increases in PD-L1 and PD-1 in whole mammary tissues during post-partum mammary gland involution; a known driver of post-partum tumor growth, invasion, and metastasis in pre-clinical models. Importantly, implantation of murine mammary tumor cells during post-partum mammary gland involution elicits a CD8+ T cell population that expresses both the co-inhibitory receptors PD-1 and Lag-3. However, upon anti-PD-1 treatment, during post-partum mammary gland involution, the involution-initiated promotional effects on tumor growth are reversed and the PD-1, Lag-3 double positive population disappears. Consequently, we observed an expansion of poly-functional CD8+ T cells that produced both IFNγ and TNFα. Finally, lymphatic vessel frequency decreased significantly following anti-PD-1 suggesting that anti-PD-1/PD-L1 targeted therapies may have efficacy in reducing tumor growth and dissemination in post-partum breast cancer patients.

## Introduction

Post-partum breast cancer patients in our cohort, or breast cancer patients diagnosed within 10 years of last childbirth, are ~3–5 times more likely to develop metastasis in comparison to non-post-partum, or nulliparous, patients (1, 2). Additionally, post-partum patients have increased LN involvement and peri-tumor lymphatic vessel density (LVD) (2–4). In 2010, Asztalos et al. identified alterations to gene expression patterns in normal mammary tissues from post-pregnant women that persist up to 10 years post-partum in comparison to nulliparous women (5). Specifically, they observed changes to genes involved in inflammation, angiogenesis, extracellular matrix (ECM), and breast cancer; suggesting that the mammary microenvironment after pregnancy may be conducive to malignancy. Consistent with this hypothesis, recent pregnancy can increase a woman's risk for developing breast cancer for more than 20 years following childbirth (6–8). Normal mammary gland development associated with pregnancy consists of a period of expansion of the mammary epithelium, to prepare the gland for lactation, followed by full differentiation of the mammary epithelium into milk secreting cells. Following lactation, or pregnancy in the absence of lactation, post-partum mammary gland involution occurs to return the mammary epithelium to the pre-pregnant state. Previous studies of normal post-partum mammary gland involution in rodents and women have revealed that attributes of this normal developmental process are similar to those observed in breast tumors (9–12). These attributes include establishment of a tissue microenvironment that is characterized by ECM remodeling, increased LVD, immune infiltration and evidence of immune suppression (3, 13–15). Additionally, non-metastatic tumor cells implanted into this tissue microenvironment in pre-clinical models grow and invade more rapidly, seed micro-metastases, and are durably altered to a more invasive and metastatic state; suggesting that post-partum involution can drive intrinsic, pro-metastatic changes, in tumor cells (3, 16). These findings predict that the process of normal involution may drive post-partum breast cancer (PPBC) metastasis.

Post-partum mammary gland involution, induced by weaning, has been extensively studied in rodents where it is characterized by two phases of tissue remodeling. The first, known as the reversible phase, is triggered by milk stasis and results in death of the secretory mammary epithelium (17, 18). The second phase, known as the irreversible phase, consists of stromal remodeling and repopulation of the gland with adipocytes. Insight into molecular programs that govern this developmental process in mice has been gained through gene expression profiling studies on whole mammary tissues where roles for death receptors and immune mediators were revealed (10, 11, 19). Additionally, influx of immunosuppressive Foxp3+ regulatory T cells and IL-10+ macrophages occurs during involution resulting in effector T cell suppression (13). Furthermore, M2-like or tissue repair type macrophages and macrophages with pro-lymphatic phenotypes are evident (3, 20, 21). As epithelial cell apoptosis during involution likely results in the increased presentation of self-antigens (22) it is not surprising that numerous cell types initiate an immune-tolerant microenvironment and, consequently, an environment that could be primed for post-partum tumor growth. Importantly, LVD also increases during mammary gland involution, presumably to promote the clearance of increased fluid—generated by milk stasis, apoptotic cell debris, and immune cell infiltrates. However, we have recently shown that the mammary lymphatics that arise during post-partum involution are also capable of transporting tumor cells to distant lymph nodes during the active phase of tissue remodeling and that post-partum patients are enriched for lymph node involvement (2, 21). These studies suggest that metastatic seeding via lymphatics may be an early event in post-partum patients, which may account for the increased metastasis observed (21).

Tumor-associated lymphatic vessels not only promote dissemination (23–27), but have recently been shown to reduce anti-tumor immune responses in tumor models (28, 29). Lymphatic endothelial cells (LECs) normally promote peripheral immune tolerance in the lymph node during homeostasis. Specifically, lymph node LECs express PD-L1 to inhibit auto-reactive T cells via engagement of the inhibitory receptor Programmed Death-1 (PD-1) (30–34). In addition, inhibitory receptor expression of PD-1 is also an important marker of T cell effector function. However, upregulation of multiple inhibitory receptors, such as PD-1, Lag-3, and TIGIT can occur as a result of chronic antigen stimulation and lead to non-responsive T cells that fail to successfully clear the pathogen (35–40). In the cancer setting, a similar phenomenon occurs as tumor-infiltrating T cells upregulate multiple co-inhibitory receptors and are limited in their ability to produce multiple effector cytokines (such as IFNγ and TNFα) (41), making them less polyfunctional and unable to clear the tumor (42). Recently, several studies have pointed to a role for tumor-associated lymphatic and/or macrophage expression of PD-L1 in contributing to T cell inhibition (28, 29, 43, 44). In addition, we and others have published that PD-L1 expression by LECs promotes their survival during an immune response and a role for PD-L1 expression in promoting tumor cell survival has been demonstrated (45–48). Thus, PD-L1 clearly plays a role in promoting cell survival and immunosuppression in multiple cell types present in the tumor microenvironment (TME).

Since T-cell infiltration, immune suppression, macrophage infiltration, and lymphangiogenesis have all been described during post-partum involution (3, 13, 21), we sought to determine whether T cell expression of co-inhibitory receptors allows for immune evasion by tumor cells during post-partum involution and whether this mechanism could be reversed with anti-PD-1 treatment. In this manuscript, we investigate the immune regulatory state of the mammary tissue during post-partum involution and in tumors implanted during involution (post-partum tumors). We demonstrate that PD-L1 and PD-1 are a part of the involution program and show increased expression of PD-L1 on lymphatic endothelial cells, and cells of myeloid lineage, as well as PD-1 on T cells during mammary gland involution and in post-partum tumors. Importantly, we demonstrate that this mechanism is an integral part of the tumor promotional program effects of involution. Administration of an anti-PD-1 antibody to mice during involution, when the tumors are established, reduced growth of the post-partum tumors to levels observed in nulliparous hosts. Upon evaluation of the tumor infiltrating CD8+ T cells we discovered co-expression of two inhibitory receptors, PD-1 and Lag-3, that were specific to tumors established during involution. Following treatment with anti-PD-1, the PD-1+Lag-3+ inhibitory CD8+ T cell population disappeared and the frequency of total CD8+ T cells and polyfunctional CD8+ T cells increased significantly suggesting a reversal of at least some of the immunosuppressive effects of involution. Surprisingly, anti-PD-1 treatment also reduced tumor/involution associated LVD suggesting this treatment may have implications for stopping lymphatic-mediated metastasis. Our results lay the ground work for additional studies aimed at uncovering the potential role of the mammary LECs during involution, and in the tumor microenvironment, in promoting immunosuppression and suggest a potential treatment option for PPBC patients.

## Materials and Methods

### Animal Studies

All animal procedures were approved by the University of Colorado Anschutz Medical Campus Institutional Animal Care and Use Committee. BALB/c and C57Bl/6 (age 6–8 weeks) were obtained from Charles River Laboratories. Mice were crossbred for involution studies; C57Bl/6 female mice were bred in triad with BALB/c males and BALB/c females were bred with C57Bl/6 males. Age-matched nulliparous females were used as controls. At 10–14 days post-parturition, post-partum mammary gland involution was instigated in the bred females by force-weaning the pups. For normal involution studies, mammary glands, and draining lymph nodes (inguinal lymph nodes) were harvested from nulliparous and involution day 6 mice. Tumor studies for the 66cl4 and E0771 mouse mammary carcinoma models were performed as previously described (21) with the tumors, mammary glands, and lymph nodes being taken for flow cytometry, immunohistochemistry, and downstream biochemical analyses. For the E0771 PD-1 intervention studies, 250,000 tumor cells were injected into the number 4 mammary glands of either nulliparous or involution day 1 C57Bl/6 dams. Tumor sites were palpated daily (E0771) or twice weekly (66cl4) for tumors. Calipers were used to take measurements and the tumor volumes were calculated using length x width x width × 0.5. Additionally, 66cl4 tumor cells were luciferase and GFP tagged allowing for tumors to be detected using the Xenogen 200. Once tumors became measurable, mice were randomized into control or treatment groups and injected with 250 μg of either isotype control (anti-IgG2a [clone 2A3; Bio X Cell cat. #BP0089]) or anti-PD-1 (clone RMP1-14; Bio X Cell cat. #BP0146) antibody, respectively. Injections were administered intra-peritoneally every third day. Tumor studies were ended based on primary tumor cell growth or ulceration at 3–4 weeks post injection (66cl4) or 1–2 weeks post injection (E0771). *In vivo* studies were performed in triplicate with pooled or representative data shown.

### Mammary Gland Processing and Staining (IHC)

Mammary glands were harvested and placed into 10% neutral buffered formalin for 48 h. After 48 h, tissues were moved to 70% EtOH, processed, and stained for LYVE-1 as previously described (3, 14, 16, 21, 49).

### Lymphatic Vessel Density Quantification

Lymphatic vessel density (LVD) was performed as previously described (3, 21). Briefly, slides stained for PDPN (D2-40) or LYVE-1 were scanned into the Aperio ImageScope software. Lymphatic vessels were counted in the tumor-adjacent tissue (peri-tumor region) and LVD was quantified as the number of lymphatic vessels per area of tissue.

### Human Tissue Acquisition

Research using de-identified human breast tissue (Supplemental Table 1) was conducted under a protocol deemed exempt from subject consent as approved by the Colorado Multiple Institution Review Board (COMIRB) and tissues were acquired by Virginia Borges as previously reported (1). Dr. Borges obtained written informed consent from the patients, the studies were conducted in accordance with recognized ethical guidelines (e.g., Declaration of Helsinki, CIOMS, Belmont Report, U.S. Common Rule), and the studies were approved by an institutional review board.

### Staining of Human Tissue Using Vectra

Four-micron thick sections were taken from Formalin Fixed Paraffin Embedded tissue, dewaxed in xylenes and rehydrated. Slides were placed in 10% NBF for 20 min for extra fixation, rinsed with DI water, then submerged in Target Retrieval Solution pH6 (Dako cat# S1699) and placed in a pressure cooker for 20 min. Slides were rinsed with Dako wash buffer (Dako cat# K8000), blocked for 10 min with Perkin Elmer Diluent/Block (Perkin Elmer cat# ARD1001EA), then sequentially stained for the following markers: PD-L1 (clone E1L3N), PD-1 (clone NAT105;), PDPN (clone D2-40), and CD68 (clone KP1). Incubation time for all primary antibodies was 1 h at room temperature. Slides were rinsed and stripped in Target Retrieval Solution in between every primary. Slides were then incubated in Perkin Elmer Opal Polymer HRP Mouse+Rabbit secondary (cat# ARH1001EA) for 30 min at room temperature, followed by a 10 min incubation in Opal Fluorophore reagents (Perkin Elmer). After the final stain, Spectral DAPI (Perkin Elmer cat# FP1490) was applied to slides for 5 min, then slides were rinsed and cover-slipped with ProLong Diamond Antifade Mountant (Thermo cat# P36970). Multispectral imaging was then performed using the Vectra 3 Automated Quantitative Pathology Imaging System (Perkin Elmer). Whole slide scans were collected using the 10x objective and 5–10 regions were selected for multispectral imaging with the 20x objective. The multispectral images were analyzed with inForm software (Perkin Elmer) to unmix adjacent fluorochromes, subtract autofluorescence, segment the tissue into lymphatic vessels and non lymphatic vessels, segment the cells into nuclear, and membrane compartments, and to phenotype the cells according to morphology and cell marker expression. Cells with a PD-L1 threshold <0.95 were classified as PD-L1 negative while cells with a value >0.95 threshold were classified as PD-L1 positive using inForm software. To quantitate PD-L1 in lymphatics a blinded observer imaged 5–10 representative fields from the peritumor region that were positive for PDPN vessels by only the PDPN channel. For PDPN we also counted PD-L1+ lymphatic vessels by adding the PD-L1 channel and counted PDPN+PD-L1+ vessels as well as PDPN+PD-L1- vessels and calculated the percent positive per case, which was then normalized to area. PD-1+ cells were also counted in the same manner as PDPN+ vessels.

### Flow Cytometry

Tumors were separated from the mammary gland. Both tumors and mammary glands were placed in six-well plates with 2 mL of Click's media without mercaptoethanol or L-glutamine (Irvine Scientific, Santa Ana, CA), where they were minced with scalpels, digested with 500 units/ml collagenase type II and IV and 20 μg/ml DNase (Worthington Biochemical Corporation, Lakewood, NJ) and incubated for 1 h at 37°C. The tissue suspension was then filtered through a 100 μm strainer and washed with Click's. The filtered cells were centrifuged at 1,400 RPM for 5 min, the supernatant was removed, and the pellet was resuspended in 1 mL FACS buffer (500 mL 1x HBSS pH 7.4, 0.1% BSA, 0.02% sodium azide, up to 1L ddH_2_O). The tumor cells were stained with BD viability 510 dye prior to staining with CD45 (clone30-F11), CD8a APC/Cy7 (clone 53-6.7) (1:400), CD4 APC or PerCp-Cy5.5 (clone RM4-5) (1:300), PD-1 FITC or BV421 (clone 29F.1A12) (1:100), Lag-3 PerCP/Cy5.5 (clone C9B7W) (1:100), and/or CD11a FITC (clone M17/4) (1:200). The mammary glands were stained with BD viability dye 510 followed by CD45 APC-Cy7 or Pacific Blue (clone 30-F11) (1:300), CD31 Pacific Blue or PerCp-Cy5.5 (clone 390) (1:200), PDPN APC or PE-Cy7 (clone 8.1.1) (1:200), CD11b Pacific Blue or PerCp or FITC (clone M1/70)(1:400), F4/80 APC, APC-Cy7, PerCP-Cy5.5 or FITC (clone BM8) (1:100), PD-L1 PE, FITC, or BV421 (clone RMP1-30 or 29F.1A12) (1:200), and EpCAM PE-Cy7 or APC-Cy7 (clone G8.8) (1:100). Flow cytometry antibodies were purchased from Biolegend (San Diego, CA). CD8 T cells were identified from live, CD3+/CD8+, where they were further characterized by their expression of PD-1 and Lag-3. Lymphatic endothelial cells were identified from live, CD45-/EpCAM-, and CD31+PDPN+. Cells were run on the DakoCytomation CyAn ADP flow cytometer (Fort Collins, CO) or FACs Canto II, acquired using Summit software or Diva Software, and analyzed with FlowJo software (Tree Star, Ashland, OR). Geometric mean fluorescence intensity (gMFI) was calculated with FlowJo software.

### Intracellular Cytokine Staining

Cells were isolated from the tissue and treated with or without (unstimulated controls) phorbol 12-myristate 13-acetate (PMA) (20 ng/ml) (Sigma, St. Louis, MO) plus ionomycin (1 ug/ml) (Sigma, St. Louis, MO) for 4–6 h at 37 degrees in the presence of 2 ug/ml of brefeldin A (Adipogen, San Diego, CO) in RPMI+2.5% FBS. Cells were then stained with CD8, CD45, CD4, CD44, PD-1, and Lag-3 (as above) and incubated at 37°C for 30 min. Following surface marker staining cells were fixed with 1% paraformaldehyde and 4% sucrose for 10 min in the dark at room temperature. Following fixation, cells were permeabilized with BD Perm Wash (BD Biosciences, San Jose CA) and stained for cytokines IFNγ (1:200) (APC; Biolegend clone XMG1.2) and TNFα (1:200) (FITC; Biolegend clone MP6-XT22). After washing cells were resuspended in FACs buffer (0.5% Bovine Serum Albumin and 0.1% Sodium Azide in PBS) and were run on an ADP Cyan. Gating was determined based on unstimulated controls. All antibodies were purchased from Biolegend (San Diego, CA).

### TCGA RNASeq Analysis

Analysis was performed on cbioportal.org TCGA Breast provisional dataset using the co-expression tool and the RNA-Seq data.

### Statistics

One-way ANOVA, unpaired *t*-test, and linear regression were run in the GraphPad Prism software, assuming normal distributions among independent samples. For **Figure 6**, Pearson and Spearman analysis was performed on cbioportal.org. *P*-values of <0.05 were deemed significant.

## Results

### PD-L1 Expression and PD-1 T Cells Are Observed in Patients With PPBC and in Pre-clinical Models

**Figure 1:**
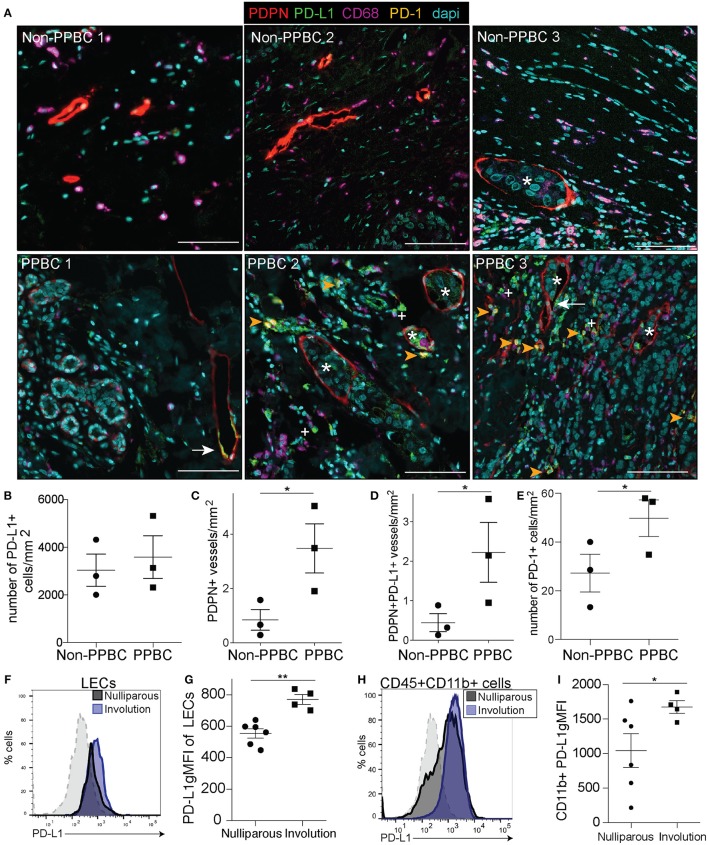
PDPN/PD-L1/PD-1 in post-partum patients and mouse mammary tumor models. **(A)** Tumor adjacent mammary tissues from three nulliparous non-post-partum breast cancer patients (Non-PPBBC) and three patients with post-partum breast cancer (PPBC) stained for PDPN (red), PD-L1 (green), CD68 (magenta), PD-1 (orange), and dapi (cyan). Asterisks indicate tumor cells found within the lymphatic vessel, white arrows indicate PD-L1+ lymphatic vessels, plus signs indicate PD-L1+ macrophages (CD68) and orange arrows indicate PD-1+ cells. **(B)** Number of PD-L1+ cells in the peritumoral region of the patients from A. **(C)** Number of PDPN+ vessels in the peritumoral region of the patients from A. **(D)** Number of PDPN+ vessels that are also PD-L1+ in the peritumoral region of the patients from A. **(E)** Number of PD-1+ cells in the peritumoral region of the patients from A. **(F)** Histogram of PD-L1 expression by LECs (CD45-EpCAM-CD31+PDPN+PD-L1+) from 66CL4 tumors implanted during mammary gland involution. Gray dotted line indicates a PD-L1 negative population (CD45+CD11b-F4/80-) that does not change. **(G)** gMFI of PD-L1 of LECs in tumor. **(H)** Histogram of PD-L1 expression by CD45+ CD11b+ cells from tumors implanted during mammary gland involution. Gray dotted line indicates a PD-L1 negative population (CD45+CD11b-F4/80-) that does not change. **(I)** gMFI calculated for PD-L1 from D. Data shown from animal experiments are from 1 representative experiment of 2 replicates with at least 4 tumors per group. Unpaired *t*-test: *p < 0.05; **p < 0.01. Scale bars are 100 microns in length.

To determine if the increased lymphatics (PDPN) that we observe in our patients with PPBC exhibit upregulation of PD-L1 and/or whether PD-1+ T cell infiltration occurs in the tumor microenvironment (TME) of PPBCs, we utilized multispectral imaging of the peri-tumor region in tissues from three different patients with PPBC, who were within 1-year (PPBC1), 3 years (PPBC2), and 4 years post-partum (PPBC3) (Supplemental Table 1). In comparison to three non-PPBC patients, who were all nulliparous, we observed increased PDPN+ LVD in the peri-tumor region from our PPBCs. We also observed frequent lymphatic vessel expression of PD-L1 (arrow) and that lymphatic vessels were frequently infiltrated with PD-L1 expressing tumor cells (asterisk), identified by their altered nuclear morphology. Furthermore, we also observed the presence of CD68+ macrophages that appear to express PD-L1 (+ symbol) as well as cells expressing PD-1 (arrowhead) in the surrounding areas (Figure 1A and Supplemental Figure 1). We quantitated PD-L1+ cells in the peritumor region and observed that total numbers of PD-L1+ cells per area did not differ between groups (Figure 1B), but that total PDPN+ vessel density, as well as PDPN+PD-L1+ vessel density, were increased in our patients with PPBC compared to nulliparous controls (Figures 1C,D) suggesting that PD-L1 lymphatics are a part of the TME in patients with PPBC. Finally, we observe a significant increase in PD-1+ cells in the TME of our PPBC patients (Figure 1E).

Since we observed PD-L1 expression in cells of the TME from patients with PPBC, we next asked if PD-L1 expression was a characteristic of the TME of post-partum tumors in our murine model of PPBC. Using the 66cl4 isograft model where we have shown increased lymph vessel density (LVD) and lymph node metastasis in post-partum hosts (3), we examined post-partum tumor-associated LEC expression of PD-L1 in Balb/c mice. As previously observed, tumor cells implanted on day 1 of involution (involution group tumors) exhibited decreased latency and increased growth compared to nulliparous (Supplemental Figure 2A) (3, 13, 16). At study endpoint, 4 weeks post-injection, tumors were harvested, and populations were analyzed by flow cytometry (Supplemental Figures 2B,C). We observed an increase in the fluorescence intensity of PD-L1 on CD45-Epcam-CD31+PDPN + tumor associated LECs (Figures 1F,G), which we also observed with implantation of E0771 tumors into BL6 mice (Supplemental Figure 3A). We found that while there were similar frequencies of the monocyte (CD11b+) populations in both tumor models (Supplemental Figure 3B and not shown), CD11b+ cells in both 66cl4 and E0771 tumors implanted during mammary gland involution had higher average levels of PD-L1 (Figures 1H,I and Supplemental Figure 3C). The number of PD-L1+ cells was also increased in the involution group, but this increase was lost when normalized to tumor size (Supplemental Table 2). We also analyzed additional cell populations for PD-L1 including monocytes, fibroblasts, blood endothelial cells (BECs), and EpCAM+ tumor cells based on described markers. We did not see significant staining differences or staining above background in the fibroblasts, BECs or EpCAM+ cells (Supplemental Figure 3D). These results complement our recently published data showing that CD11b+ monocytes in the TME of involution group tumors contribute to lymphangiogenesis and extend our observations to describe their expression of PD-L1 (21).

**Figure 2:**
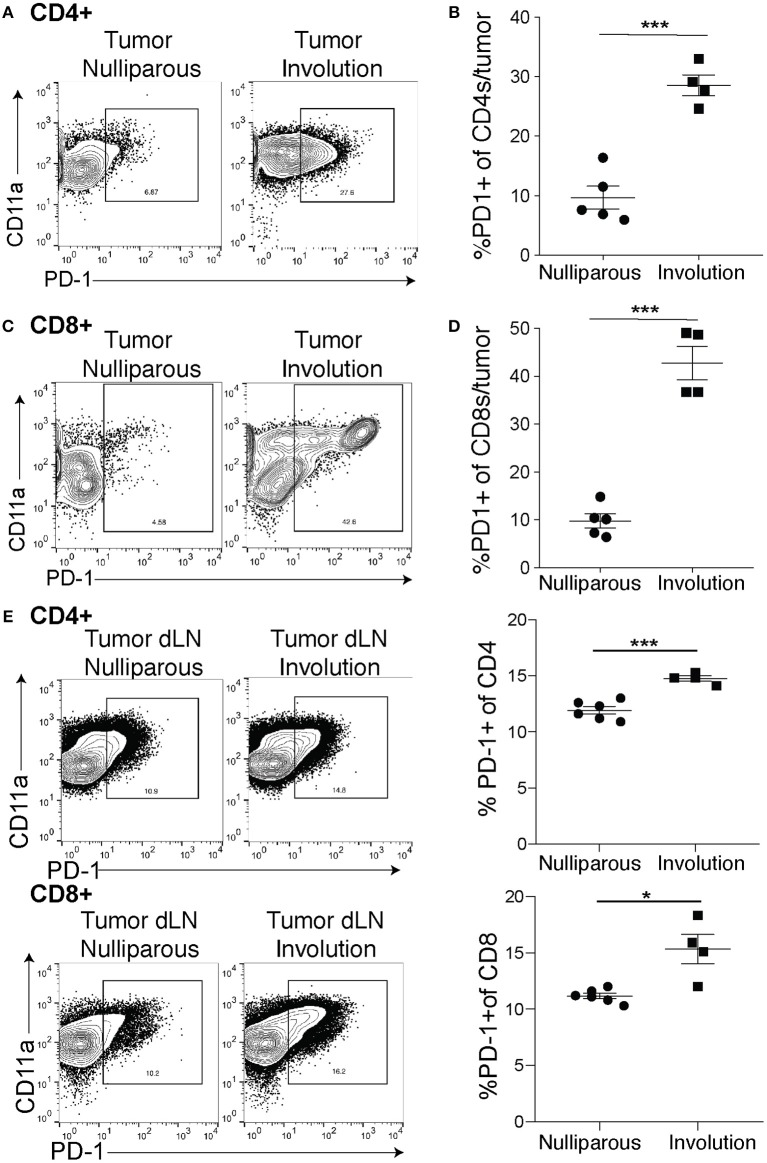
Tumor-associated PD-1^+^ T cells are increased in tumors implanted during involution. **(A)** Representative flow plots for cells from nulliparous or involution group tumors of CD11a and PD-1 expression by CD4^+^ T cells. **(B)** Quantification of CD4^+^ T cells that express PD-1 from A. **(C)** Representative flow plots of CD8^+^ T cell expression of PD-1 and CD11a from nulliparous and involution tumors and **(D)** quantification of PD-1^+^ CD8^+^ T cells. **(E)** Representative flow plots of CD11a and PD-1^+^ CD4^+^ T cells or CD8^+^ T cells in the tumor draining lymph nodes and quantification of PD-1+ frequency of each cell type. Data shown are from 1 representative experiment of 2 replicates with at least 4 tumors per group. Unpaired *t*-test: *p < 0.05; ***p < 0.001.

As PD-L1 is the inhibitory ligand, we next examined expression of the inhibitory receptor, PD-1, by CD4+ and CD8+ tumor-associated T cells (gating-Supplemental Figure 2D) all of which are also positive for CD11a, a molecule that has been shown to render them unable to control tumor growth (50). We found an increase in the expression of PD-1 on CD4+ T cells (Figure 2A) and after quantification we found that an average of 30% of CD4+ T cells in the involution group tumors expressed PD-1 compared to 10% in the nulliparous controls (Figure 2B). As cytotoxic CD8 T cells are typically thought to be important in controlling tumors we next asked about expression of PD-1 on CD8+ T cells. We observed a striking difference in the expression profile of CD8+ T cells in tumors implanted during mammary gland involution compared to tumors implanted in nulliparous hosts (Figure 2C). When we quantified this difference, we found a >4-fold increase in expression of PD-1 by CD8+ T-cells from involution group tumors (Figure 2D). Additionally, we observed similar phenotypes in the mammary draining lymph nodes of tumor bearing animals (Figure 2E) suggesting this mechanism of tumor-associated immune suppression may extend beyond the local tumor microenvironment to drive the increased LN metastasis that we observe in post-partum patients. Finally, we observed similar phenotypes in the E0771 tumors, but not lymph nodes (Supplemental Figures 4A,B).

### PD-L1 and PD-1 Expression Are Observed in Mouse Mammary Tissues During Normal Post-Partum Mammary Gland Involution

**Figure 3:**
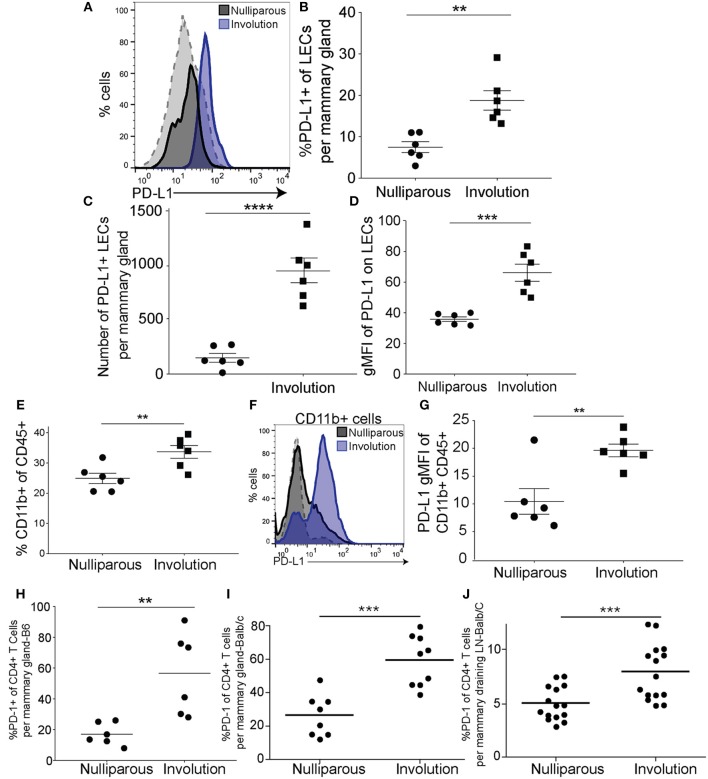
PD-L1^+^ lymphatic endothelial cells and PD-L1^+^ myeloid cells increase in the mammary gland during post-partum involution. **(A)** Representative histogram of PD-L1 by lymphatic endothelial cells (CD45^−^EpCAM^−^CD31^+^PDPN^+^PD-L1^+^) acquired by flow cytometry from B6 mammary glands of nulliparous (*n* = 6) or recently weaned, involution day 6 (*n* = 6), mice. Gray dotted line indicates a PD-L1 negative population (CD45+CD11b-F4/80-) that does not change. **(B)** Quantification of the frequency of PD-L1+ LECs of total LECs per mammary gland. **(C)** Number of PD-L1^+^ LECs per mammary gland. **(D)** Quantification of gMFI of PD-L1 expression by LECs from A. **(E)** Frequency of CD11b^+^ cells of the CD45^+^ cells found in the mammary gland in nulliparous or involution group mice. **(F)** Histogram of PD-L1 expression by CD11b^+^ cells in mammary gland of nulliparous or involution group mice. Gray dotted line indicates a PD-L1 negative population (CD45^+^CD11b^−^F4/80^−^) that does not change. **(G)** Quantification of PD-L1 expression by gMFI on CD11b+ cells as shown in F. **(H)** Percent of CD4^+^ T cells that are PD-1^+^ from the mammary glands of B6 mice and **(I)** as in H except from Balb/c mice. **(J)** Percent of PD-1^+^ CD4^+^ T cells in the axillary lymph nodes from nulliparous and involution group tissues. Data shown are from 1 representative experiment of 2 replicates with at least 6 mammary glands per group. Unpaired *t*-test: **p < 0.01; ***p < 0.001; ****p < 0.0001.

We next asked if the LECs of C57/BL6 (B6) or Balb/c female mice exhibit expression of PD-L1 during normal post-partum mammary gland involution, similar to what is observed in LN LECs to induce peripheral tolerance during tissue homeostasis (31). We evaluated mouse mammary LECs at involution day 6, the peak of the remodeling phase of involution, for expression of PD-L1. PD-L1+ LECs were evaluated based on the markers described above and validated by staining with isotype and fluorescence minus one (FMO) controls (Supplemental Figure 2B). We observed that expression of PD-L1 was increased in mammary LECs isolated from involution group mice compared to nulliparous (Figure 3A). We also found that the percentage and number of LECs expressing PD-L1, as well as the geometric mean fluorescence intensity (gMFI) of PD-L1, was increased on LECs during involution (Figures 3B–D), which is similar to that observed in our pre-clinical model of PPBC (Figures 1F,G). We found these increases in both the B6 mice as well as the Balb/C mice (Supplemental Figures 5A–C). Since we observed increased PD-L1 expression in the CD11b+ population in our model of PPBC, we first confirmed that the CD11b+ population increased (gating-Supplemental Figure 2C) during involution in both B6 (Figure 3E) and Balb/C (Supplemental Figure 5D) mouse mammary glands. Then, we also observed an increase in expression of PD-L1 by this population (Figure 3F) that was significant (Figure 3G) in both models (Supplemental Figure 5E).

To better understand if PD-1 expression on T cells is similarly increased during post-partum mammary gland involution, we evaluated the T-cell compartment at involution day 6 by assessing the frequency of PD-1 expression by CD4+ and CD8+ T-cells in B6 and Balb/c mice compared to isotype controls (gating-Supplemental Figure 2D). Similar to previous results in Balb/c mice (51), we observed a significant increase in the percent of PD-1+ CD4+ T cells in mammary glands from B6 (Figure 3H) and confirmed this in our Balb/c mice (Figure 3I). This increase in PD-1+ CD4+ T cells also extended to the LN (Figure 3J). Further, we found an increase in CD8+ T cells expressing PD-1 in the B6 mice (Supplemental Figure 5F), which was not significant in the Balb/c mice (Supplementary Figures 5G,H). These results suggest that a mechanism of LEC and/or monocyte/macrophage mediated T cell inhibition could be driving the decreased latency and increased growth rate that we observe in our pre-clinical models when tumor cells are implanted during involution (16, 21, 52).

### PD-1 Targeted Therapy Reduces Tumor Growth in Post-partum Hosts by Reactivating T-Cells

**Figure 4:**
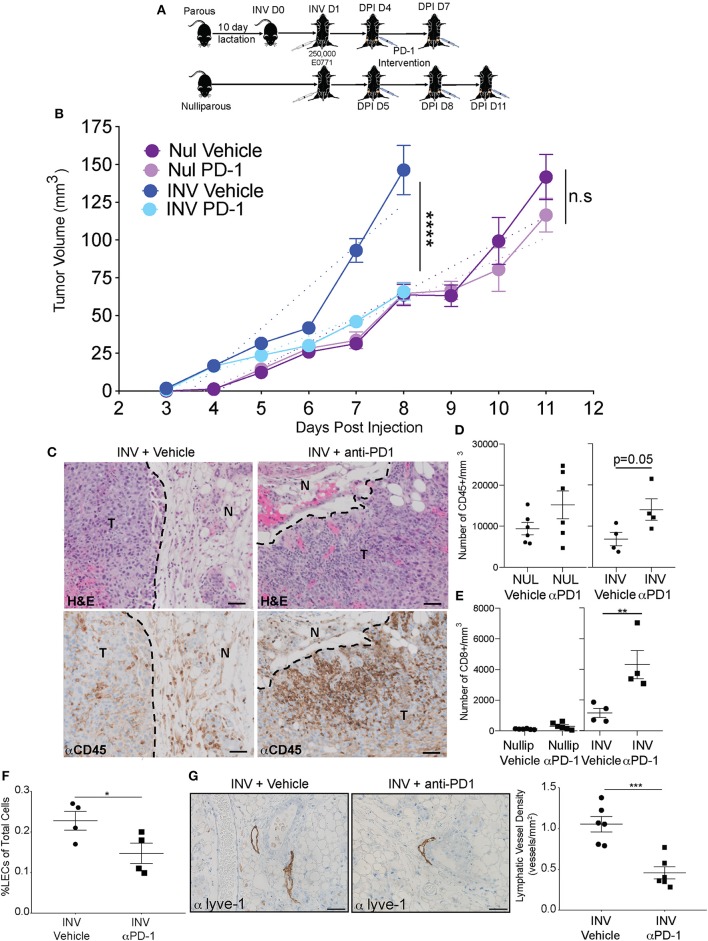
Anti-PD-1 treatment reduces tumor growth, enhances immune infiltration, and reduces lymphatic vessels in a model of post-partum breast cancer **(A)** C57/Bl6 mice were bred and allowed to lactate for 10 days before pups were removed to initiate mammary gland involution (parous/involution, *n* = 6); age-matched nulliparous controls were used (*n* = 6). Parous animals were injected with 250,000 E0771 tumor cells at Inv D1 (involution group) or into nulliparous animals (nulliparous group). Once tumors became measurable (involution = 4 days post injection (DPI); nulliparous=DPI D5), anti-PD-1 intervention was administered and continued every third day. E0771 mammary tumor growth curves from nulliparous and involution group C57Bl/6 mice treated with vehicle or anti-PD-1 are shown in **(B)**. Results are representative from two independent studies. Dotted lines represent the slope of the tumor growth. **(C)** Representative images of H&E analysis and immunohistochemistry for CD45 (brown) in fixed tumor tissue to identify tumor infiltrating lymphocytes after anti-PD-1 treatment compared to vehicle controls, T = tumor and N = normal. CD45^+^ area was 9.99% of tumor area for involution with vehicle and 22.62% of tumor area for involution with anti-PD-1 treatment. Scale bars are 50 microns. Number of **(D)** CD45^+^ and **(E)** CD8^+^ cells per area in tumors from B quantified by flow cytometric analysis. **(F)** % LECs of total in tumors from B quantified by flow cytometry. **(G)** Representative images of Lyve-1 stained fixed tumor adjacent tissues and quantitation of Lyve-1^+^ vessels per area in involution group tumors +/- PD-1 treatment. Scale bars are 100 microns. *p < 0.05; **p < 0.01, ***p < 0.001, ****p < 0.0001.

A prediction of our results is that inhibition of PD-L1/PD-1 signaling during involution would dampen the increased tumor growth observed when tumors are implanted during involution by reversing this involution-driven mechanism of immune suppression. To test our hypothesis, we utilized the E0771 mouse mammary tumor model since tumors implanted in this model become palpable during active involution; when the immune suppressive mechanism is most activated. Thus, we orthotopically implanted E0771 tumor cells into the intact mammary glands of C57BL/6 mice at involution day 1 or into nulliparous hosts. Then, we blocked PD-L1-mediated inhibition of T cells by administering an anti-PD-1 monoclonal antibody every third day after tumors were palpable and size-matched in both nulliparous and involution groups. Following two and three treatments, in involution and nulliparous mice, respectively, mice were euthanized and flow cytometry performed on tumors (Figure 4A). Similar to previous results, involution group tumors exhibited decreased latency and increased growth compared to tumors in nulliparous hosts (Figure 4B) (3, 13, 16). Importantly, the anti-PD-1 treatment significantly reduced the growth rate in involution group tumors, to levels more similar to those observed in the nulliparous hosts, and did not significantly affect tumor growth in the nulliparous group (Figure 4B). We then evaluated whether the anti-PD-1 treatment affected immune cell infiltration into the tumors in the involution group. Immunohistochemistry (IHC) on a single tumor from each involution group revealed increased intratumoral staining for CD45, which was validated by our flow cytometry where we observed a >2-fold increase in the number of CD45+ cells in the tumor (Figures 4C,D). We also observed a significant increase in the number of intra-tumoral CD8+ T cells following treatment with anti-PD-1 when the tumors were implanted into involution hosts (Figure 4E). Additionally, we found a significant decrease in LEC frequency in the adjacent mammary tissues of involution group mice treated with anti-PD-1 by both flow cytometry (Figure 4F) and by tissue staining with PDPN to assess LVD (Figure 4G). Conversely, we found no significant difference in PD-L1 expression by LECs after anti-PD-1 treatment (Supplemental Figure 6A). These findings suggest that blockade of PD-1 could have potential for blocking both tumor growth and lymphogenous tumor cell spread during involution.

**Figure 5:**
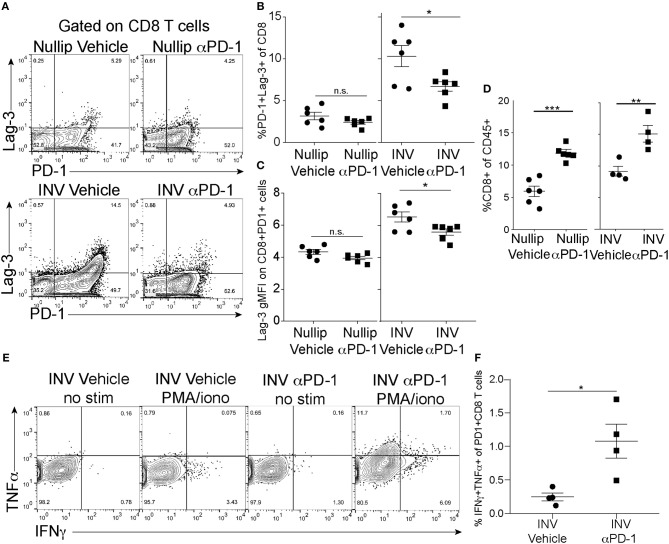
Tumor Infiltrating T cells after PD-1 treatment. **(A)** Example flow cytometry plots of CD45^+^CD3^+^B220^−^CD8^+^ T cells from each treatment group. Shown is expression of Lag-3 and PD-1. **(B)** Frequency of CD8^+^ T cells that express both PD-1 and Lag-3 as shown in A-upper right quadrant. **(C)** gMFI of Lag-3 on PD-1^+^ CD8^+^ T cells (upper right quadrants in A). **(D)** Frequency of CD8+ of CD45+ T cells in tumors treated with anti-PD-1 as measured by flow cytometry. **(E)** Flow cytometry plots of CD45^+^CD3^+^CD8^+^PD-1^+^ cells at end of experiment (cells from right quadrants from C) after PMA/ionomycin treatment and intracellular cytokine staining with IFNγ and TNFα. **(F)** Quantification of double positive IFNγ and TNFα cells from upper right quadrant in G in involution group tumors with or without treatment. Data shown are from 1 representative experiment of 2 replicates with at least 4 tumors per group. Two-way ANOVA (A). Unpaired *t*-test **(C–G)**: *p < 0.05; **p < 0.01; ***p < 0.001.

To evaluate whether anti-PD-1 treatment was affecting the phenotype and functionality of the tumor-associated T cells, we used flow cytometry to assess the co-expression of co-inhibitory markers, PD-1 and Lag-3, by the CD8+ T cells (Figure 5A) as well as PD-1 single positive expression by the CD8+ and CD4+ T cells (Supplemental Figures 6B,C). With treatment, we observed a decrease in the frequency of PD-1 and Lag-3 double positive cells in the involution group tumors, but not in the nulliparous tumors (Figure 5B). Importantly, the Lag-3 gMFI was also significantly decreased following anti-PD-1 treatment in the involution group, but not the nulliparous, (Figure 5C) and this effect was not due to the treatment antibody (clone RMP1-14) blocking the staining antibodies (clone RMP1-30 or 29F1A12) (Supplemental Figure 6D). We also observed that the frequency of the CD45+ cells that were also positive for CD8 was increased in involution group tumors, and the nulliparous, that were treated with anti-PD-1 (Figure 5D). However, the number of CD8+ cells was not increased (see Figure 4E) nor was tumor growth significantly affected by anti-PD-1 treatment in the nulliparous group (Figure 5D). While PD-1 and Lag-3 are markers of T cells exhaustion, these markers do not evaluate the functionality of the T cells. Therefore, we next measured the production of the effector cytokines IFNγ and TNFα by tumor-associated CD8+ and CD4+ T cells in our involution group tumors. We evaluated CD8+PD-1+ T cells *ex vivo* in the presence or absence of stimulation with PMA/Ionomycin (Figure 5E). After anti-PD-1 treatment we found significant increases in the production of both IFNγ and TNFα by CD8+PD1+ T cells, from the involution group treated with anti-PD-1, suggesting that they are poly-functional (Figure 5F). The frequency of CD4+ T cells and the production of IFNγ by CD4+ T cells after anti-PD-1 treatment was not significantly different with treatment (Supplemental Figures 6E,F).

### Co-expression of Immune Inhibitory Programs and PD-L1, PDPN, and CD68 Is Observed in Patients With Breast Cancer

**Figure 6:**
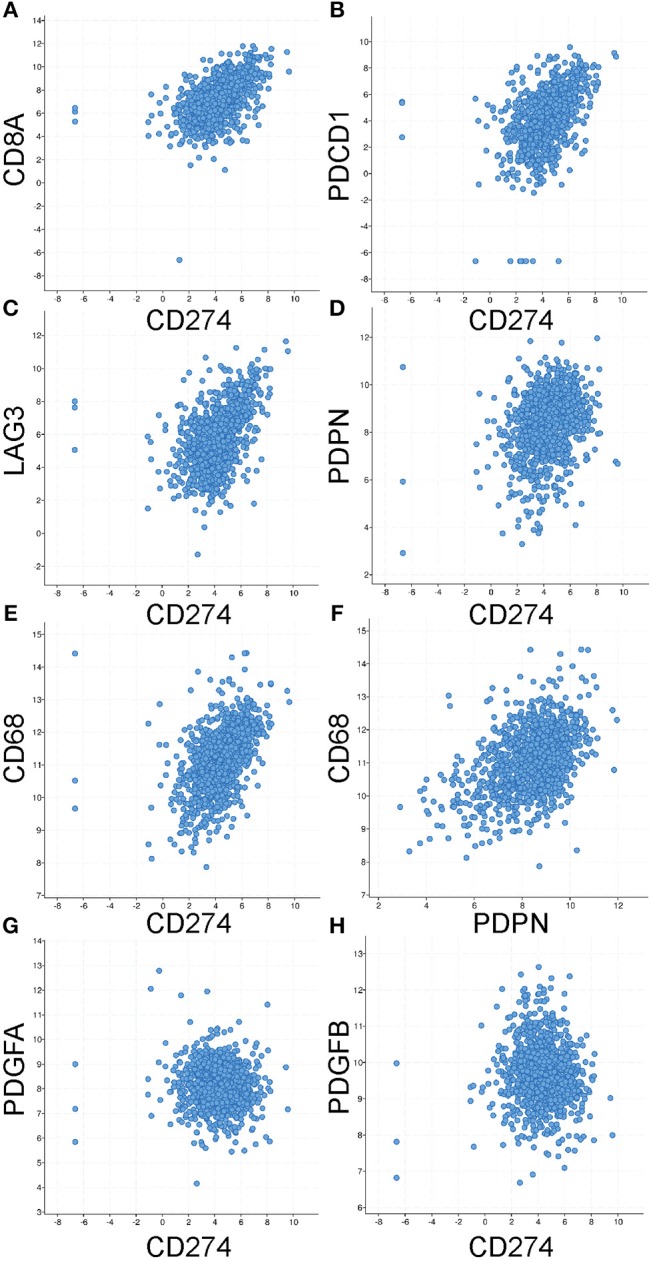
Co-expression of identified markers of immune suppression in primary breast cancers. Using the co-expression tool in cBioPortal to analyze mRNA (by RNASeq) levels of multiple markers reveals significant positive correlations between CD274 (the gene encoding for PD-L1) and **(A)** CD8A **(B)** PDCD1 (the gene encoding for PD-1) **(C)** LAG3 and **(D)** PDPN and **(E)** CD68. As well as between PDPN and CD68 **(F)**. No correlation was observed with **(G)** PDGFA, a marker of fibroblasts, or its heterodimeric partner PDGFB **(H)**. Statistical analyses are provided in Table 1.

Having shown that PD-L1+ LEC and monocytic cell populations likely contribute to the immune inhibitory microenvironment in the mammary gland during involution and in breast cancer, we examined whether breast cancer patient samples frequently exhibit co-expression of immune-inhibitory programs and of the LEC marker PDPN and the macrophage marker CD68. To accomplish this, we examined co-expression by RNASeq in breast cancers using The Cancer Genome Atlas cBioPortal for Cancer Genomics. As expected we observed co-expression of PD-L1 (gene name *CD274*) with CD8 (gene name *CD8A*), PD-1 (gene name *PDCD1*), and *LAG3* (Figures 6A–C). We also observed significant co-expression of *CD274* with *PDPN* and *CD68* as well as between *PDPN* and *CD68* (Figures 6D–F). Correlation coefficients and *p*-values for each relationship are reported in Table 1. Furthermore, consistent with our results during in mouse mammary tissue during involution we did not observe significant co-expression of CD274 with *PDGFA*, an established fibroblast marker, or with *PDGFB* the heterodimeric partner of *PDGFA*. Therefore, we predict that this mechanism of immune suppression is mediated, in part, by cells of the TME may be at play in breast cancer patients and that analysis of LEC and monocyte/macrophage content could be utilized to predict whether a breast cancer patient is likely to respond to PD-1-targeted therapy.

**Table 1:** Statistics for mRNASeq correlation analyses.

**Genes**	**Spearman**	***p-value***	**Pearson**	***p-value***
CD274	0.55	1.33e−88	0.51	3.06e−75
CD8A				
CD274	0.51	4.39e−73	0.45	1.00e−56
PDCD1				
CD274	0.49	6.50e−68	0.45	3.64e−57
LAG3				
CD274	0.26	5.92e−19	0.27	1.7e−19
PDPN				
CD274	0.52	1.68e−77	0.47	1.86e−62
CD68				
PDPN	0.42	4.98E−48	0.45	2.13E−55
CD68				
CD274	−0.05	0.100	−0.05	0.0849
PDGFA				
CD274	−0.08	5.746Ee−3	−0.04	0.216
PDGFB				

## Discussion

Our results suggest that an immune inhibitory microenvironment involving LEC and monocyte/macrophage expression of PD-L1 and T-cell expression of PD-1 is induced during involution and may be co-opted by post-partum tumors. These inhibitory receptor-ligand interactions appear to promote increased tumor growth and immune evasion by increasing PD-1 and Lag-3 expression by tumor infiltrating T cells. In 2017, Dieterich et al. published that LECs in the tumor microenvironment could promote T cell exhaustion through expression of PD-L1 (28) and more recently Lane et al. identified a mechanism by which T cell infiltration can manipulate PD-L1 expression via IFNg (29). These results are similar to normal mechanisms of T-cell inactivation by LECs in the lymph node where LEC expression of peripheral tissue antigens and PD-L1 act to prevent autoimmunity and promote peripheral tolerance (30, 31, 33). Here, we suggest that a similar mechanism is induced during post-partum mammary gland involution by showing that PD-L1+ LECs and monocyte/macrophage populations and PD-1+ T-cells are abundant. Additionally, we believe that both increased lymphatics and their expression of PD-L1, along with increased PD-1 and Lag-3 expression by T cells in the tumor microenvironment could lead to increased tumor metastasis in tumors established during involution. We also show that specific targeting of the PD-L1/PD-1 interaction in our model of PPBC decreases mammary tumor growth during involution. Identification of the mechanisms that underlie the increased expression of co-inhibitory ligands and receptors during involution, and in post-partum tumors, is important for proposing rational clinical trials for post-partum patients.

We predict that the immune suppressive environment, which occurs as a consequence of mammary gland involution and is mediated, in part, by lymphatics and monocyte/macrophage populations, aids in prevention of autoimmunity in a tissue healing environment (3, 10, 13, 20, 53, 54). While our studies do not identify the specific pathways activated in the mammary gland during involution that control PD-L1 expression by LECs or monocytes/macrophages, and other cells, several possibilities are evident from the existing literature. First, pro-inflammatory enzyme cyclooxygenase-2 (COX-2) is expressed and active during involution, and correlations between COX-2 expression and PD-L1 expression have been reported in lung cancer, melanoma, a mouse model of mammary cancer, and in tumor-associated macrophages and myeloid derived suppressor cells (55–59). Additionally, blocking COX-2 during involution also decreases some of the tumor-promotional effects of involution including increased growth (16). Second, STAT-3 is activated during involution and is a known activator of PD-L1 expression (60–65). Third, high expression of PD-L1 in the subcapsular sinus LECs in the lymph node (30, 45) occurs through lymphotoxin beta receptor (LTBR or TNFRSF3) signaling and is mediated by the presence of B cells (30). Interestingly, LTBR signaling is also a known regulator of epithelial cell apoptosis during involution (19) and B cells are increased in the mammary gland during involution (13) suggesting an additional mechanism that may drive LECs to upregulate PD-L1 expression. We propose that a number of mechanisms could be at play to result in the upregulation of PD-L1 on lymphatic endothelial cells, as well as other cells, in the mammary gland during mammary gland involution and in post-partum tumors. As we have previously published that macrophages contribute to lymphatic remodeling, and lymphatic mimicry, during involution it is notable that the monocyte/macrophage population expressing PD-L1 is also higher during involution and in tumors implanted during involution. We suggest that this increase in myeloid cells could either be to remodel the lymphatic vasculature or to drive expression of PD-L1 through as of yet undefined mechanisms. It is also possible that the increased PD-L1 myeloid population is contributing to the expression of PD-L1 by incorporating into the vasculature and expressing lymphatic markers. Dissecting these molecular mechanisms that drive PD-L1 expression is under active investigation.

In addition to increased PD-L1 expression, we show that tumor infiltrating T cells express more co-inhibitory receptors in mammary tumors implanted during post-partum involution. We also found that treatment with anti-PD-1 in these tumors resulted in decreased frequencies of PD-1 and Lag-3 double-positive CD8+ T cells and decreased Lag-3 expression. The frequency and expression of Lag-3+ cells in anti-PD-1-treated involution group tumors was more similar to nulliparous group mice, suggesting that anti-PD-1 treatment could reverse the immune suppression observed in involution group tumors by decreasing or inhibiting the expansion of Lag-3 and PD-1 double positive T cells. An alternative explanation is that the anti-PD-1 depletes PD-1+ T cells and this possibility is being explored through complementary anti-PD-L1 studies by our group. Further, while IFNγ expression is relatively low in bulk tissue during involution, we do see IFNγ expression by T cells in mammary tumors implanted into both nulliparous and involution group hosts demonstrating that IFNγ may at least partially contribute to PD-L1 expression by the cells in the tumor (29). These findings indicate that while the production of IFNγ within the tumor is not increased, that instead the frequency and poly-functionality (IFNγ and TNFα double positive cells) of CD8+ T cells is increased following treatment with anti-PD-1. These findings predict increased tumor killing consistent with what is seen in patients (41) and is consistent with the decreased tumor volume we observed. Our findings that LVD is also decreased after anti-PD-1 treatment could either be a cause or consequence of tumor regression, but is likely due to the treatment induced pro-inflammatory environment in contrast to the anti-inflammatory environment observed in post-partum tumors. Loss of LVD could significantly impact the ability of tumor cells to migrate to the tumor draining lymph node, which could also reduce metastasis. A limitation of our current studies is the lack of metastatic data in our preclinical models as well as the lack of analysis of PD-L1 expression on post-partum tumors; these important studies are being actively pursued using additional models.

Our findings that an involution-targeted therapy, anti-PD-1, can mitigate the decrease in tumor growth and LVD that we observe after implantation during involution are consistent with our previous results showing that inhibition of COX-2 can decrease growth, invasion, LVD, and metastasis (3, 16). Whether anti-PD-1 therapy could also mitigate the increased metastasis observed in our models is unanswered by our current data. However, if such mechanisms are maintained long term, as is suggested by our 66cl4 model where tumors are isolated long after completion of involution, post-partum patients with metastasis may also benefit from anti-PD-1 therapy, which has shown efficacy in the metastatic setting (see below). Our findings may also lend insight into the increased aggressiveness and metastatic potential of breast cancer diagnosed within 5–10 years of recent childbirth. We propose that a plausible mechanism is immune suppression in both the mammary tissue and the draining lymph node, which could account for the increased lymph node positivity observed in post-partum patients (2). This, along with the increased lymphangiogenesis and lymphatic vessel invasion observed in the adjacent mammary tissue of post-partum patients, could provide a favorable route for metastatic spread. While further studies addressing mechanisms of lymphatic expression of PD-L1 and lymphatic growth in tumors must be performed, we believe these studies provide substantial evidence that current immunotherapies could benefit patients with PPBC. Immunotherapy, specifically anti-PD-1/PD-L1 based, has been investigated in breast cancer with positive results. First, pembrolizumab—the highly selective monoclonal-antibody-based therapy against PD-1—was the first shown to be successful as a monotherapy for metastatic triple-negative cases of breast cancer (TNBC), with some long-lasting responses reported, and has also shown benefit for advanced ER+/Her2-in the KEYNOTE trials (66–70). It is also currently in clinical trial with several chemotherapy partners, including in the neoadjuvant setting and as a single drug in the post-neoadjuvant setting for patients with residual cancer (71, 72). Anti-PD-L1 based therapy, specifically atezolizumab, is now a standard of care option in combination with the chemotherapy drug nab-paclitaxel for TNBC that have at least 1% PD-L1 expressing tumor infiltrating immune cells, based on the results of the Impassion 130 clinical trial, which demonstrated a 10-month improvement in overall survival for the combination (73). While the early immunotherapy trials with check-point block inhibitors have met with success, many patients do not benefit or do not achieve long-term benefit and death due to progressive cancer remains the norm. This highlights that additional markers, such as evaluation of LVD, may be better or synergistic with existing markers for predicting patient response (74). Importantly, several trials are ongoing to investigate the use of anti-PD-1 and anti-PD-L1 in the (neo) adjuvant setting and the treatment has proven safe and well-tolerated with preliminary results demonstrating promising efficacy (71, 72, 74). Here we have identified a specific population of patients who may benefit from (neo) adjuvant PD-1/PD-L1 blockade—women diagnosed within 10 years post-partum who are at high risk for metastasis. Ongoing research is investigating this possibility as well as whether a combined approach with an anti-lymphangiogenesis-based therapy could improve survival for PPBC patients.

## Ethics Statement

This study was carried out in accordance with the recommendations ethical guidelines (e.g., Declaration of Helsinki, CIOMS, Belmont Report, U.S. Common Rule) of the Colorado Multiple Institution Review Board (COMIRB) committee with written informed consent from all subjects. All subjects gave written informed consent in accordance with the Declaration of Helsinki. The protocol was approved by the COMIRB. This study was carried out in accordance with the recommendations of the Guide for the Care and Use of Laboratory Animals, Animal Welfare Act and PHS Policy by the University of Colorado Anschutz Medical Campus Institutional Animal Care and Use Committee. The protocol was approved the institutional animal care and use committee.

## Author Contributions

BT and TL designed and executed experiments, analyzed results, and drafted the manuscript. AE, JF, and AW performed experiments, analyzed data, and critically reviewed the manuscript. VW performed imaging experiments. VB provided samples and critically reviewed the manuscript.

### Conflict of Interest Statement

The authors declare that the research was conducted in the absence of any commercial or financial relationships that could be construed as a potential conflict of interest.

## Abbreviations

LEC: Lymphatic endothelial cell
PDPN: Podoplanin
PD-1: Programmed Death-1
PD-L1: Programmed Death-Ligand 1
Lag-3: Lymphocyte-activation gene-3
LN: Lymph node
LVD: Lymphatic Vessel Density
TME: Tumor Micro Environment.
