# Supplementary material for: PD-1 Blockade During Post-partum Involution Reactivates the Anti-tumor Response and Reduces Lymphatic Vessel Density
Source: Front Immunol. 2019 Jun 11;10:1313. doi: 10.3389/fimmu.2019.01313 (PMC6579890; doi:10.3389/fimmu.2019.01313)
Supplement: Supplementary file 1 [file Data_Sheet_1.PDF]

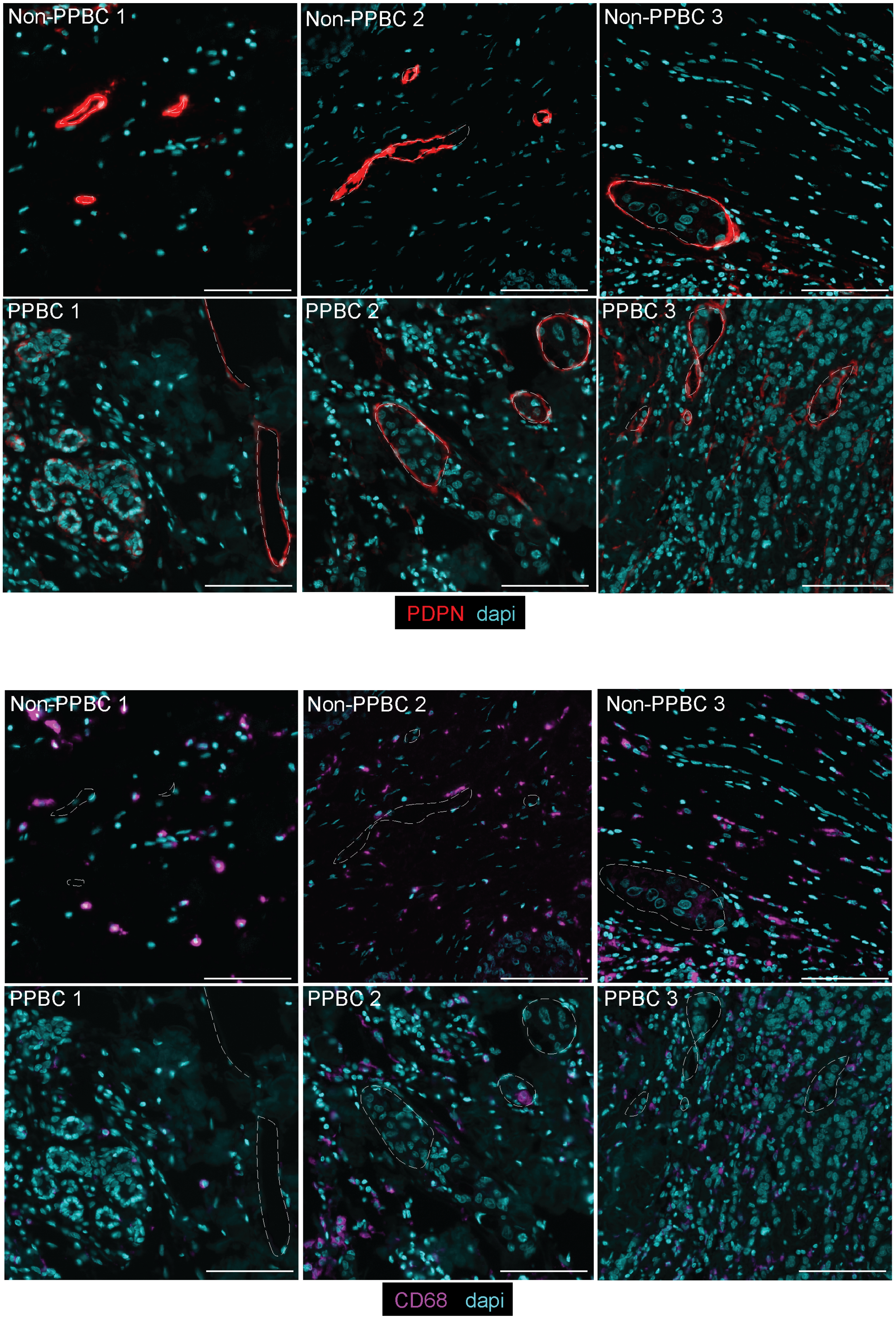
Non-PPBC 1
Non-PPBC 2
Non-PPBC 3
PPBC 1
PPBC 2
PPBC 3
PDPN dapi
Non-PPBC 1
Non-PPBC 2
Non-PPBC 3
PPBC 1
PPBC 2
PPBC 3
CD68 dapi

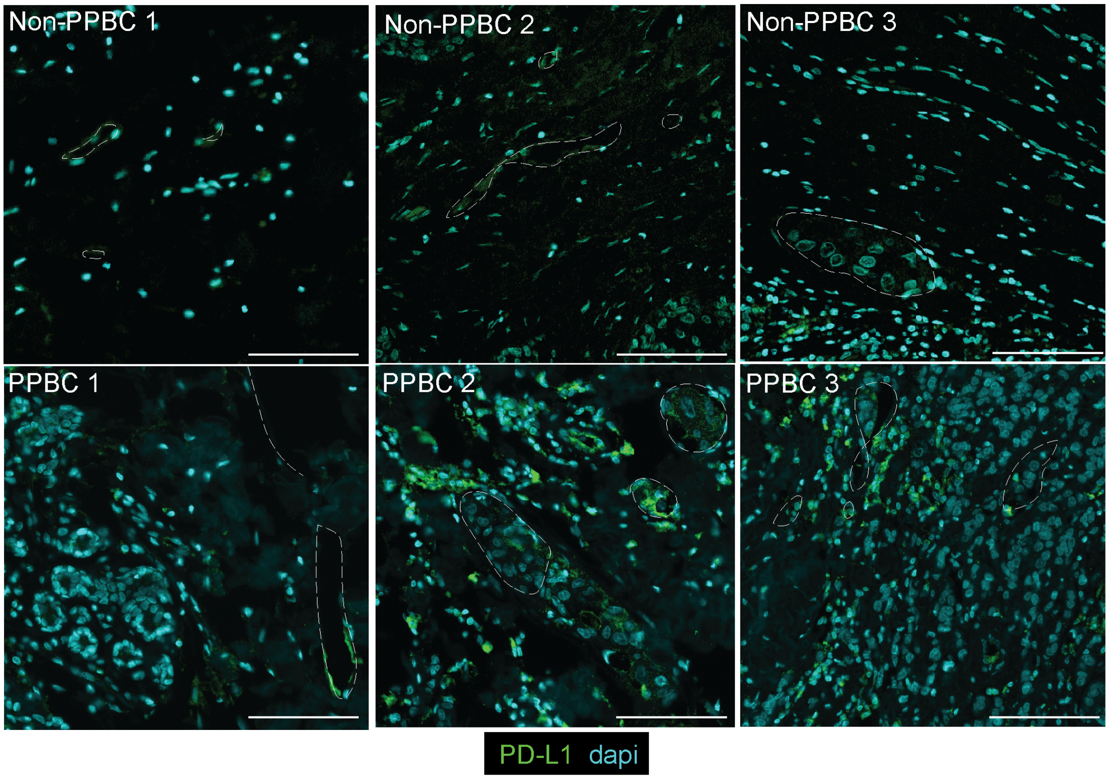

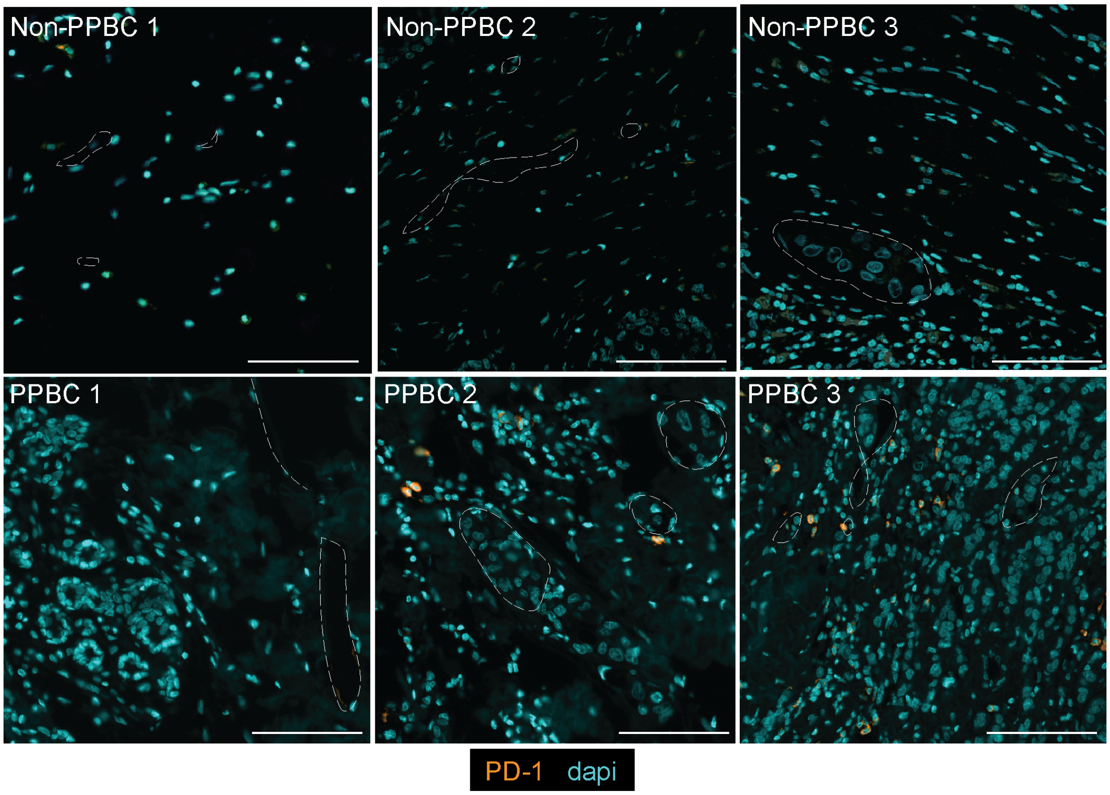

Supplemental Figure 1. Nulliparous or Postpartum Breast Cancer single color controls. Patients described in figure 1 were stained as described in figure 1. Shown are single colors for each antibody used for each section along with DAPI to visualize nuclei. **(A)** PDPN single color in red. **(B)** CD68 single color in magenta with PDPN+ lymphatic vessel outlined with white dotted line. **(C)** As in B with PD-L1 in green. **(D)** As in B with PD-1 in orange. Scalebars = 100 uM and dotted lines indicate location of lymphatic vessels based on PDPN staining in part 1A.

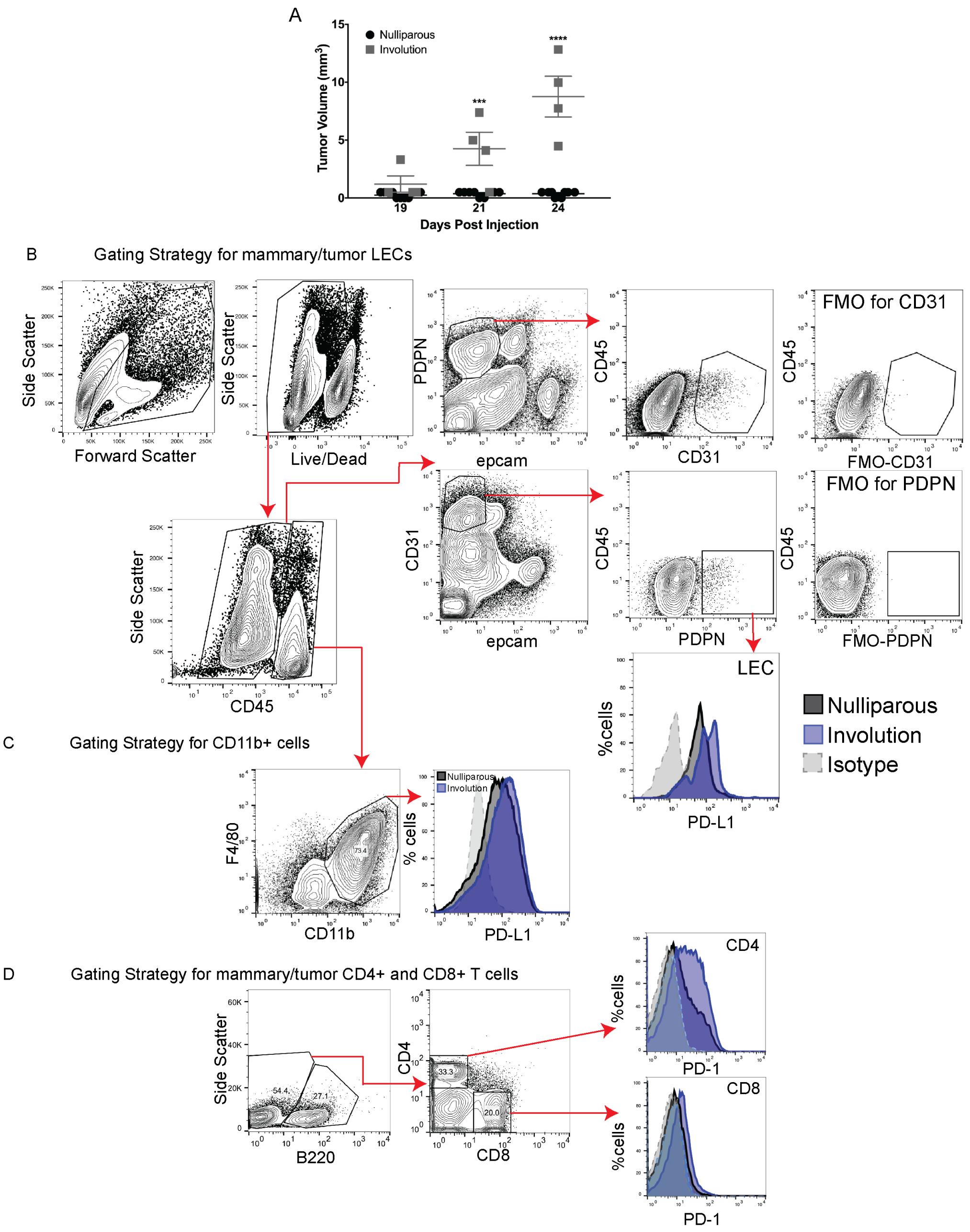

Supplemental Figure 2. Gating strategy for Flow Cytometric analysis of mammary glands and tumors. (A) Tumor volume measurements at day 19 or 21 and 24 from 66cl4 tumor cells implanted into nulliparous or involution day 1 mice. Unpaired t-test: p<0.01; ***, p<0.001; ****, p<0.0001. **(B)** Gating strategy to evaluate lymphatic endothelial cells (LEC) in the mammary gland or tumor. Live cells were gated using the BD Viability Dye 510. To gate LECs CD45- cells were gated and EpCAM-, PDPN+, and CD31+ cells were gated. Shown are the Fluorescence minus one (FMO) where all colors but CD31 were used or all colors but PDPN were used to demonstrate how LECs were distinguished. CD45-EpCAM-PDPN+CD31+ cells were then evaluated for PD-L1 expression compared to an isotype control for PD-L1. Arrows indicate which groups are shown. **(C)** Gating strategy for monocytes/macrophages. CD11b+ cells were gated and then PD-L1 expression was determined. **(D)** Gating strategy for CD4 and CD8 T cells in the mammary gland and tumor. T cells were gated as live and CD45+ as in A, then B220- and CD4 or CD8+. Surface markers or cytokine expression were then evaluated see Figure 5.

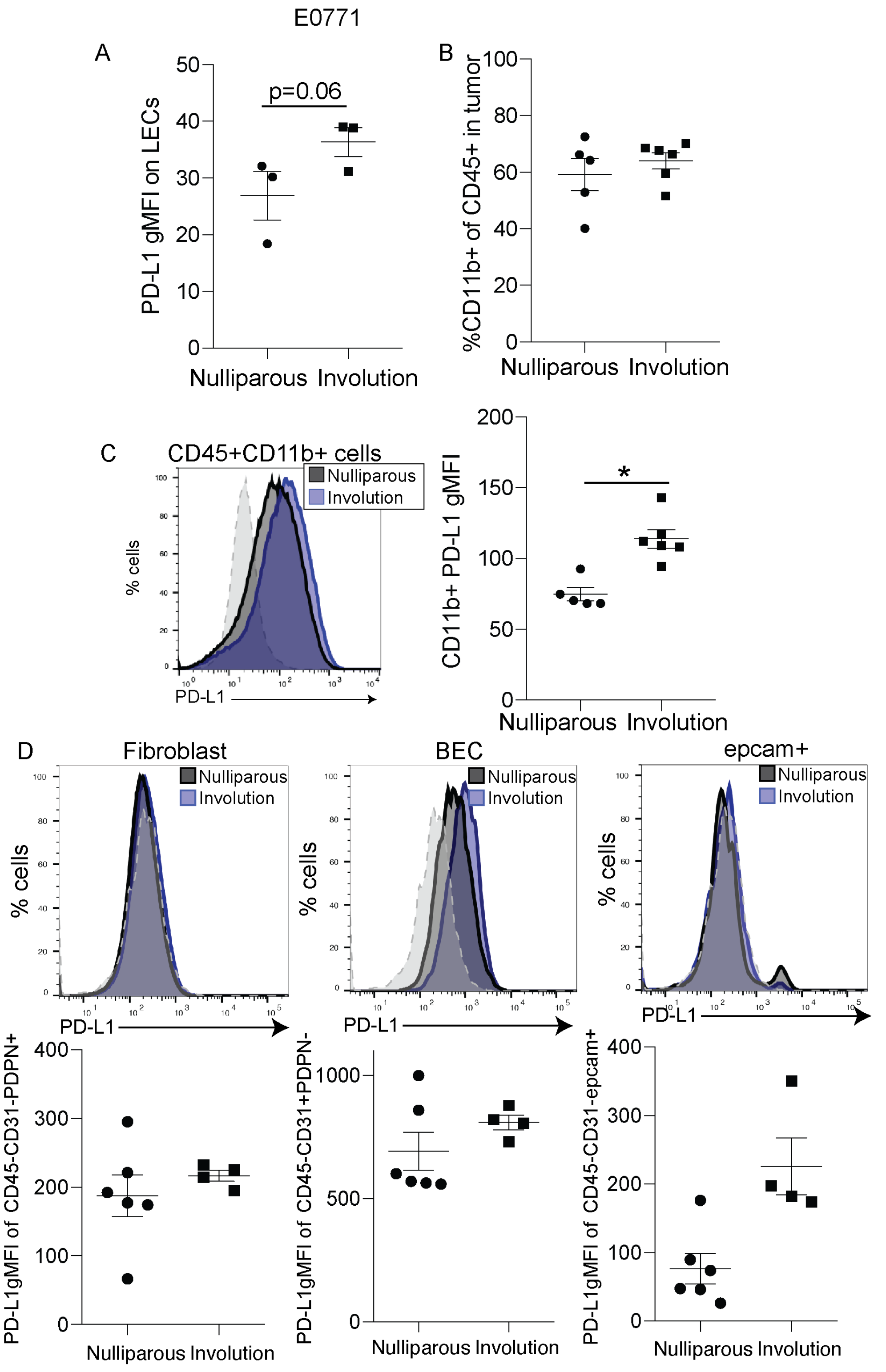

Supplemental Figure 3. PD-L1 expression on additional cell types. A) Expression level, by gMFI, in E0771 tumor associated PD-L1+ LECs. **(B)** The frequency of CD11b+ cells of CD45+ cells was calculated as well as PD-L1 gMFI as in figure 1D&E. **(C)(top)** Representative histograms of PD-L1 expression on fibroblasts, blood endothelial cells (BECs), and EpCAM+ cells from isolated from 66cl4 tumors. **(bottom)** Quantification of PD-L1 gMFI and markers used to identify fibroblasts, BECs, and EpCAM+ cell populations.

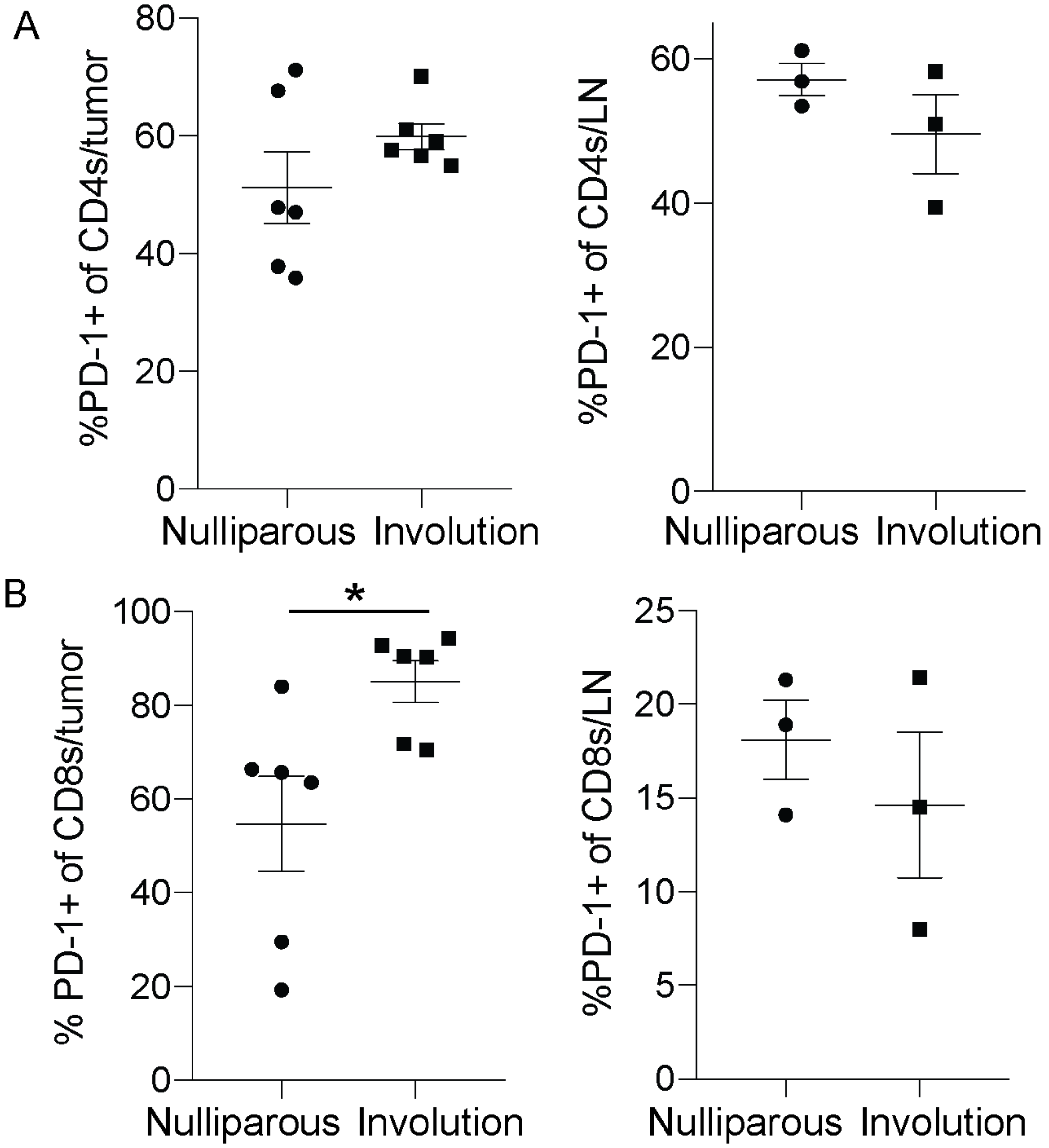

Supplemental Figure 4. E0771 mammary tumors evaluated PD-1 expression. (A) Percent of PD-1+ CD4+ T cells per tumor or tumor draining lymph node were calculated as in figure 3. **(B)** As in C except with CD8+ T cells. Unpaired t-test: *, $p<0.05$; **, $p<0.01$; ***, $p<0.001$; ****, $p<0.0001$. Experiments were completed at least twice with at least 4 tumors with similar results.

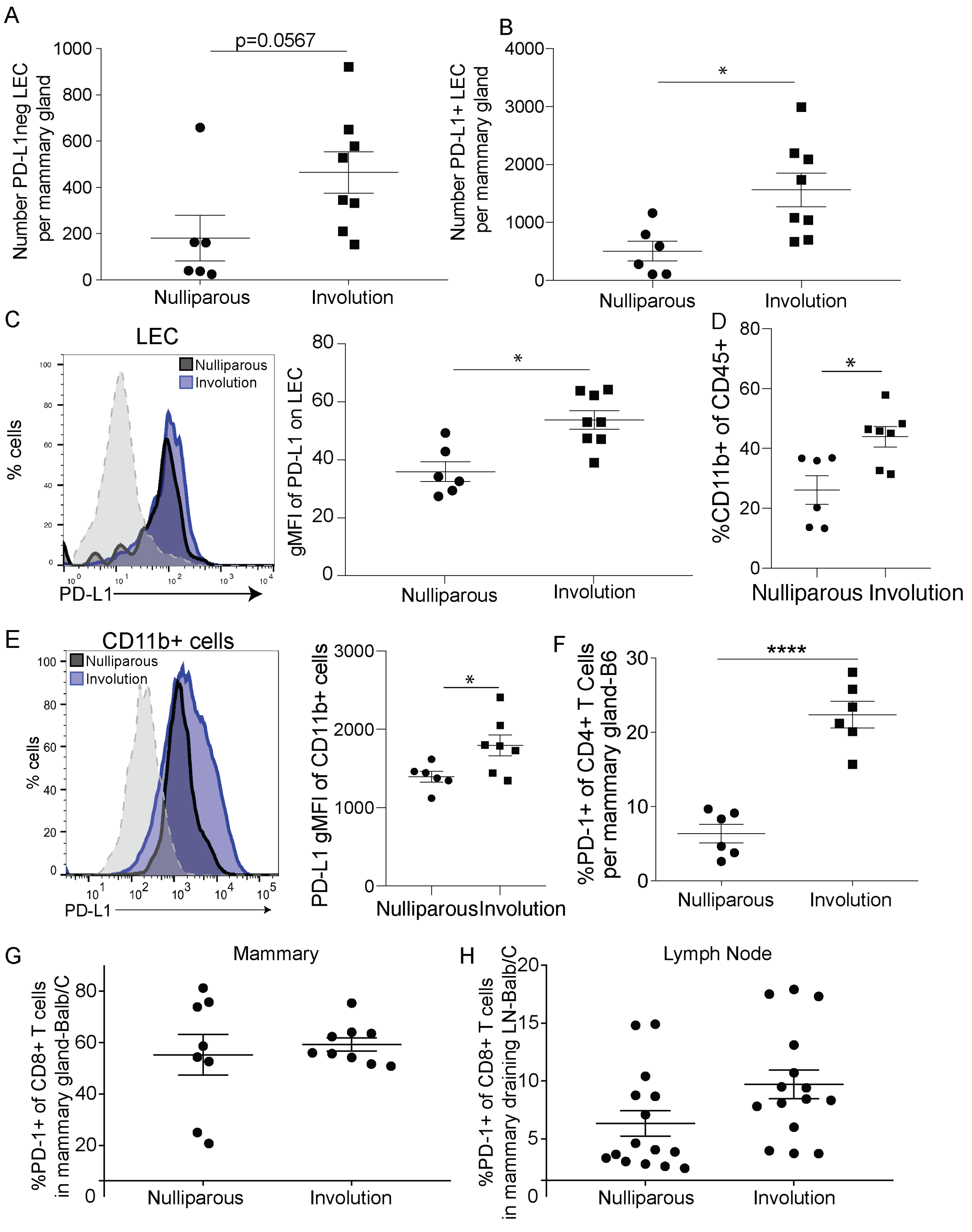

Supplemental Figure 5. PD-L1 and PD-1 expression of immune and lymphatic endothelial cells in mouse mammary glands during involution. (A) Number of PD-L1- LECs in Balb/c mammary glands. **(B)** Number of PD-L1+ LECs in Balb/c mammary glands. **(C)** gMFI of PD-L1 expression by LECs in Balb/c mammary glands. **(D)** Frequency of CD11b+ cells of CD45+ cells in Balb/c mammary glands. **(E)** gMFI of PD-L1 expression by CD11b+ cells in Balb/c mammary glands. **(F)** Frequency of PD-1+ cells of CD4+ T cells per mammary gland in B6 mice. **(G)** Percent PD-1+ CD8+ T cells per Balb/c mammary gland or **(H)** lymph node. Unpaired t-test: *, p<0.05; **, p<0.01; ***, p<0.001; ****, p<0.0001. Experiments were completed at least twice with at least 6 tumors with similar results.

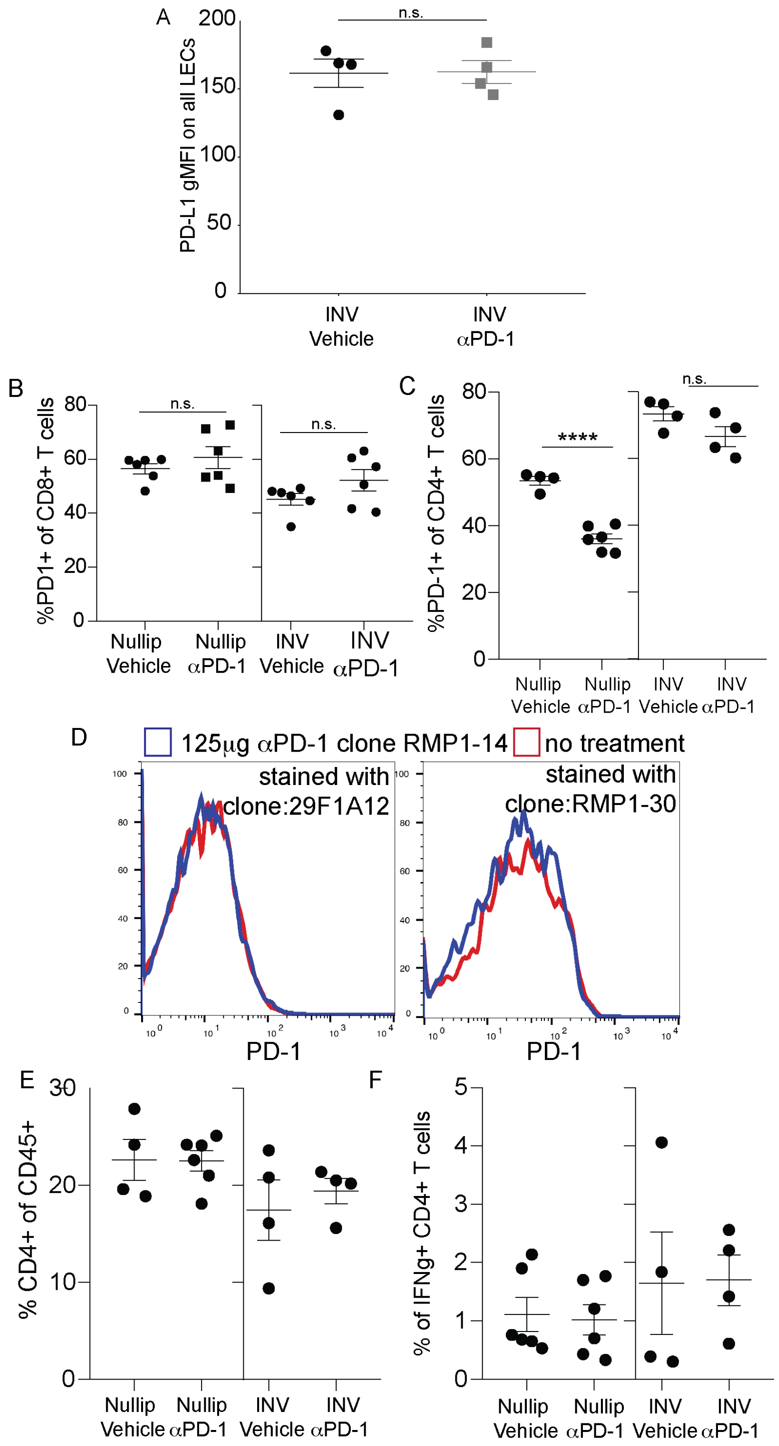

Supplemental Figure 6. T cell phenotype and function in nulliparous and involution E0771 tumor hosts after anti-PD1 treatment . (A) gMFI of PD-L1 on LECs from tumors treated with anti-PD1 (INV αPD-1) or not (INV Vehicle). **(B)** Frequency of PD-1 expressing CD8+ or **(C)** CD4+ T cells from tumors implanted into nulliparous or involution day 1 mammary glands and treated with anti-PD-1 when palpable as in figure 4. **(D)** gMFI of PD-1 stained with anti-PD-1 clone 29F1A12 or RMP1-30 after pre-treatment with 125μg per ml of anti-PD-1 antibody (RMP1-14). **(E)** Frequency of CD4+ cells of CD45+ cells in indicated tumor. **(F)** Tumor cells were isolated and treated with PMA/ionomycin for 4 hours in brefeldin A. Following surface marker staining and fixation cells were permeabilized and stained for cytokine, IFNg. Shown is the frequency of IFNg+ of CD4+ T cells in each treatment group. Unpaired t-test (A,B): *, p<0.05; ***, p<0.001; ****, p<0.0001. Experiments were completed at least twice with at least 4 tumors with similar results.

Patient	Age	Years since last childbirth	Subtype	LVI?	LN mets?	PDPN+/mm2	PDPN+PDL1+/mm2
Non-PPBC 1	33	NA	Lum A	Y	Y	0.29	0.13
Non-PPBC-2	31	NA	Lum B	N	N	0.66	0.32
Non-PPBC-3	31	NA	Lum B	UNK	Y	1.58	0.88
PPBC1	36	<1 (1 month)	Lum B	Y	Y	5.04	3.57
PPBC2	33	3	Lum B	Y	Y	1.9	0.95
PPBC3	29	4	Her2	Y	Y	3.49	2.14

Supplemental Table 1. Patient characteristics for Figure 1.

	Nulliparous	Involution
n	5	4
Tumor Volume	0.5+/-0	8.755+/-1.763
Freq. of PD-L1+ LEC of Total	0.0566+/-0.0215	0.0595+/-0.0089
Total number PD-L1+LEC	189.5+/-55.197	2782+/-1355
LEC number/mm^3	379	317.76
Freq. of CD4+PD1+ of Total	0.6672+/-0.197	0.4703+/-0.235
Freq. of CD8+PD1+ of Total	0.506+/-0.166	0.8392+/-0.419
Total number CD4+PD1+	4321.34+/-2690	15857.16+/-10107
CD4 number/mm^3	8642.68	1811.21
Total number CD8+PD1+	3621.75+/-2605	28851.39+/-18345
CD8 number/mm^3	7243.5	3295.42

Supplemental Table 2. Quantification of indicated cell numbers per 66cl4 tumor volume from supplemental figure 1A.
